# Combined Traction Force–Atomic Force Microscopy Measurements of Neuronal Cells

**DOI:** 10.3390/biomimetics7040157

**Published:** 2022-10-08

**Authors:** Udathari Kumarasinghe, Lucian N. Fox, Cristian Staii

**Affiliations:** Department of Physics and Astronomy, Tufts University, Medford, MA 02155, USA

**Keywords:** neuron, axonal growth, traction force microscopy, atomic force microscopy, cellular mechanics, tissue engineering

## Abstract

In the course of the development of the nervous system, neuronal cells extend (grow) axons, which navigate over distances of the order of many cell diameters to reach target dendrites from other neurons and establish neuronal circuits. Some of the central challenges in biophysics today are to develop a quantitative model of axonal growth, which includes the interactions between the neurons and their growth environment, and to describe the complex architecture of neuronal networks in terms of a small number of physical variables. To address these challenges, researchers need new experimental techniques for measuring biomechanical interactions with very high force and spatiotemporal resolutions. Here we report a unique experimental approach that integrates three different high-resolution techniques on the same platform—traction force microscopy (TFM), atomic force microscopy (AFM) and fluorescence microscopy (FM)—to measure biomechanical properties of cortical neurons. To our knowledge, this is the first literature report of combined TFM/AFM/FM measurements performed for any type of cell. Using this combination of powerful experimental techniques, we perform high-resolution measurements of the elastic modulus for cortical neurons and relate these values with traction forces exerted by the cells on the growth substrate (poly acrylamide hydrogels, or PAA, coated with poly D-lysine). We obtain values for the traction stresses exerted by the cortical neurons in the range 30–70 Pa, and traction forces in the range 5–11 nN. Our results demonstrate that neuronal cells stiffen when axons exert forces on the PAA substrate, and that neuronal growth is governed by a contact guidance mechanism, in which axons are guided by external mechanical cues. This work provides new insights for bioengineering novel biomimetic platforms that closely model neuronal growth in vivo, and it has significant impact for creating neuroprosthetic interfaces and devices for neuronal growth and regeneration.

## 1. Introduction

Neurons are the primary cells of the brain. During the development of the nervous system neurons grow axons and dendrites, which extend to other cells and form complex networks that transmit electrical and chemical impulses throughout a living organism. Over the past decade it has become clear that the biomechanical properties of neurons (i.e., how the cells deform, respond and recover from external physical forces), as well as the cell–substrate interactions play essential roles in brain development and the formation of neuronal networks. For example, neurons generate forces to grow axons, which in turn sense their environment, process information, adapt their growth dynamics, and respond to external stimuli [1,2,3,4,5,6,7,8]. Biomechanical parameters of neurons (elastic modulus, stiffness, tension along the axon, cellular volume) play an essential role in maintaining the mechanical stability of the cell, the rearrangement of the cytoskeleton, the formation of stress fibers, and the translation of mechanical stimuli into intracellular response [9,10,11,12,13,14]. However, many of these parameters, as well as the biomechanical interactions between neurons and the surrounding environment, have not been directly measured, nor do we know the details of how these interactions change the dynamics of the internal cellular components, and thus of the cell physiological function and health.

To date, despite important recent advances, researchers still lack a fully quantitative description of growth dynamics, which incorporates the physical interactions between the neuron and the surrounding environment. In particular, there are still numerous basic unanswered questions about the mechanisms that determine neuron biomechanical behavior, such as neuron-substrate interactions, cellular response to various external cues, and how these interactions affect the formation and function of neuronal networks [1,2,3,4,5,6,7,8,9,10,11,12,13,14,15,16,17,18,19,20,21,22]. A detailed knowledge of these interactions is essential, because these stimuli together control the wiring of neuronal circuits and their function, from growth to homeostasis and cellular health. In addition, neurons are critical cells in the body that pose major challenges in terms of regeneration due to trauma and disease. For example, one of the main challenges in the fields of biomimetic design and neural repair is how to control and modulate nerve growth towards specific outcomes—biological or bioengineered interfaces and neuroprosthetic devices [1,2,3,4,5].

On a more fundamental level, a central problem in biophysics is finding the basic principles that govern axonal dynamics, and in particular describing the formation of neuronal networks in terms of a small number of physical variables. To tackle these challenges, researchers need new experimental techniques for measuring biomechanical interactions with very high force and spatiotemporal resolutions. In our previous work, we combined fluorescence microscopy with Atomic Force Microscopy (AFM) to produce systematic, high-resolution fluorescence and elasticity maps for several types of neuronal cells, including cortical, embryonic Dorsal Root Ganglia (DRG), and P-19 (mouse embryonic carcinoma stem cells) [12,13,14]. We measured how the elastic modulus of neurons changes both during axonal outgrowth and upon disruption of microtubules and actin inside the cell. Our results show reversible local stiffening of the cell during axon growth and that the increase in local elastic modulus is due to the formation of microtubules and actin filaments [12]. These experiments also demonstrate that cortical and P-19 neurons have similar elasticity maps with typical average values of 0.2–1 kPa. In contrast, DRG neurons are stiffer than P-19 and cortical cells, yielding elastic moduli with typical average values above 1 kPa [12,13]. Our recent work has investigated how the biomechanical properties of neurons are related to the internal structural components of the cell, and how these properties change in response to external stimuli from the environment: external forces, changes in temperature, and disruption of the cytoskeleton through chemical treatment of the cell [14]. These measurements demonstrate that the regions of the cell soma with high values of the elastic modulus are associated with the two main components of the cytoskeleton: actin and microtubules. We have also reported combined AFM and fluorescence experiments, in which we measured the relationship between ambient temperature, cellular volume, and elastic modulus for cortical neurons [13,14]. Our experiments show an increase by a factor of 2 in the cell elastic modulus as the temperature decreases from physiological (37 °C) to room (25 °C) temperature. Remarkably, we also found that the soma volume increases by a factor of 1.3 as the ambient temperature increases from 25 °C to 37 °C [14]. This is a significant discovery, given that most of the reported mechanical measurements for neurons (and other types of cells) have been previously performed at room temperature, and not in physiologically relevant conditions. The combined AFM/fluorescence experiments show that changes in cellular volume and elastic modulus are greatly reduced if the activity of molecular motors inside the neurons is suppressed via treatment with Taxol and Blebbistatin, two substances that are known to inhibit the dynamics of the cell cytoskeleton [12,13,14,22]. These results demonstrate that the observed variations of these biomechanical parameters with temperature are due to changes in the activity of molecular motors, which in turn induce changes in the cytoskeletal network. We have also proposed a simple theoretical model based on semiflexible chains of polymer networks, that accounts for the experimental observations [13,14].

In this paper, we perform combined atomic force microscopy (AFM), fluorescence microscopy (FM), and traction force microscopy (TFM) experiments to investigate neuron biomechanical properties and cell–substrate interactions, and to measure cellular response to external stimuli. TFM is the preferred method for measuring cell–substrate interactions, and for determining traction stresses and forces generated by motile cells [23,24,25,26,27]. In TFM, a mechanically compliant substrate is deformed by cells and the resulting displacements are measured using fluorescent beads embedded in the substrate. Previous TFM experiments have shown that neurons apply traction forces in the course of axonal growth that result in increased tension along the axons [28,29,30,31]. Here we extend the experimental capabilities of the AFM by incorporating TFM and develop a novel AFM-TFM system that allows us to measure neuron mechanical properties and biomechanical interactions between neurons and the growth substrates at very high resolution. We show that each technique gives complementary information about the biomechanical processes involved: AFM—changes in cell elasticity map of the neuron; TFM—cell–substrate interaction via traction forces and stresses; FM—cell–substrate adhesion points as well as cell internal structure (cytoskeleton, actin). In this way variations in neuron elastic modulus and cell deformations can be directly related to forces and traction stresses applied by the cell on the surface. To our knowledge, these are the first experiments combining TFM and AFM that are reported for any type of cell. Our results have significant impact for bioengineering new substrates to stimulate neuronal repair and regeneration.

## 2. Materials and Methods

### 2.1. Substrate Preparation, Cell Culture, Fluorescence and Atomic Force Microscopy Measurements

In this work, we used cortical neurons obtained from day 18 embryonic rats. The brain tissue isolation protocol was approved by Tufts University Institutional Animal Care and Use Committee in accordance with the NIH Guide for the Care and Use of Laboratory Animals. For cell dissociation and culture, we used established protocols reported in our previous work [12,13,14,15,16,17,18,19,20,21,22]. Cortical neurons were cultured on poly acrylamide (PAA) substrates coated with poly D-lysine (PDL), at a density of 5000 cells/cm^2^. Neurons cultured at relatively low densities (in the range 3000–7000 cells/cm^2^) are optimal for investigating axonal growth on surfaces with different mechanical, geometrical and biochemical properties, as previously reported [15,16,17,18,19,20,21,22]. The average elastic modulus measured by AFM for the PAA substrates used in this work was *E_S_* = (450 ± 50) Pa. These substrates have a significant deformation under the traction forces exerted by cells and are therefore suitable for TFM measurements of neurons [23,24,25,26,27,28,29,30,31]. For traction force measurements, FluoSphere bead solution (200 nm diameter, 660 nm emission, Invitrogen, Waltham, MA, USA) was added to PAA hydrogels at 5% volume. More details about the TFM experiments are given in Section 2.3.

All neurons were imaged using optical, fluorescence microscopy and AFM. Fluorescent measurements were performed on the inverted Nikon (Micro Video Instruments, Avon, MA, USA) Eclipse Ti optical stage, integrated with the Asylum Research AFM using either the 20× or 40× objectives. For actin staining, the cells were treated with 10% formalin and maintained at either 25 °C or 37 °C for 15 min. The samples were permeabilized with 0.1% Triton-100 (Sigma-Aldrich, St. Louis, MO, USA) in phosphate-buffered saline (PBS, Life Technologies, Grand Island, NY, USA) for 10 min, then incubated at room temperature for 20 min in 150 nM Alexa Fluor^®^ 564 Phalloidin (Life Technologies, Grand Island, NY, USA), and then rinsed with PBS. We acquired fluorescence images using a standard Texas Red filter: excitation: 596 nm; and emission: 615 nm.

Neuronal cells and the growth substrates were imaged using an Asylum Research MFP-3D-Bio AFM (Asylum Research, Santa Barbara, CA, USA) equipped with a BioHeater closed fluid cell, and an inverted Nikon Eclipse Ti optical microscope (Micro Video Instruments, Avon, MA, USA). To acquire the force maps on the neurons, each sample was mounted in an Asylum Research Bioheater chamber with 1 mL of cell culture medium. The samples were maintained at a constant temperature of 37 °C during the experiment, for a minimum of 15 min before starting each measurement, and no longer than 2.5 h in total. To obtain high-resolution AFM maps of the cell elastic modulus, we used the force–volume mode of the AFM [12]. We acquired 16 × 16 µm maps of individual force vs. indentation curves on each neuron, with a resolution of 1 µm between points, as previously reported [12,13,14]. We used Olympus Biolever cantilevers (Asylum Research, Santa Barbara, CA, USA) possessing a nominal spring constant of 0.03 N/m and half angle *α* = 30°, as well as stiffer cantilevers with spring constant of 2.8 N/m (Bruker AFM Probes, Camarillo, CA, USA). The value of the elastic modulus at each point on the sample was determined by fitting the force vs. indentation curves with the Hertz model for a 30° conical tip [14]. A typical force point has a fitting error of maximum 20%. The values measured for the elastic modulus at every point are reproducible within this error [12,13,14].

### 2.2. Axonal Growth and the Generation of Traction Forces

**Figure 1:**
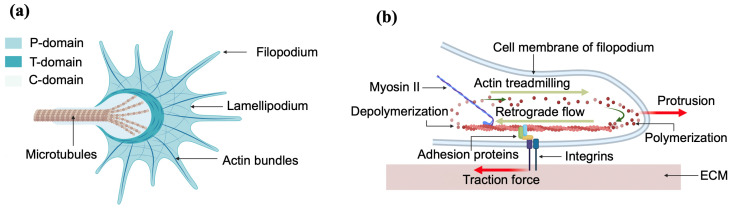
(**a**) Top-view schematic of the growth cone showing the structural components: actin bundles, microtubules, filopodia and lamellipodia; (**b**) side-view schematic of the growth cone showing the generation of traction forces through the interaction between integrins, adhesion proteins and actin bundles.

Axonal elongation is guided by the growth cone, a dynamic sensing unit located at the leading edge of the axon. The growth cone consistently follows specific routes through a complex and changing environment by responding to multiple environmental guidance cues such as extracellular matrix proteins (ECM), biomolecules released by neighboring neurons, electrical signals, substrate stiffness and geometrical cues [1,2,3,4,5,6,7,8,9,10]. Figure 1 shows the schematic diagrams and the main components of the growth cone.

Cells exert physical forces on the surrounding environment (neighboring cells, growth substrate, ECM tracks). Various cell functions such as cell signaling, proliferation, ion channel regulation, migration, morphogenesis, ECM remodeling and cell–cell interactions are also regulated in many cases by biomechanical interactions [9,10,11]. These cellular forces can be generated outside of the cell, referred to as exogenous, or generated inside of the cell and then transmitted out, known as endogenous [23]. The tangential force exerted by cells on the ECM substrate is called the traction force, and is generated inside the cell (endogenous). These endogenous traction forces are generated by the collective work of stress fibers, actin filaments, myosin, and other proteins that helps to anchor the cell to the surrounding ECM, and then transmitted to the ECM substrate through focal adhesions [1,2,3]. In the case of neurons, the generation of traction forces occurs when the growth cone adheres to the substrate through adhesion proteins and integrin-containing focal adhesions (Figure 1b). These molecules control the polymerization of actin filaments, which in turn push forward the leading end of the P domain of the growth cone (Figure 1). We will describe this process in more detail in Section 4.

### 2.3. Traction Force Microscopy

The first observation of traction forces for cells was reported by Harris et al. in 1980. The researchers observed the formation of wrinkles on a thin silicon oil layer due to the force exerted by a single cell [24]. Since then, many methods have been developed over time to quantitatively measure traction forces: Traction Force Microscopy (TFM), cantilevers and micropillar deformations, droplets and inserts, molecular sensors technologies, monolayer stress microscopy, laser ablation, and geometric force inference [25]. Among all these methods, TFM has become the main technique used to measure traction forces for cells on substrates with known mechanical properties, as it has several key advantages: it is relatively straightforward to set up, perform and interpret; and it can access a wide range of length and force scales [23,24,25,26,27].

The basic working principles of TFM are the measurement of substrate deformation on the basis of the traction forces of the cells and the calculation of the traction force using the known mechanical properties of the substrate. Fluorescent beads are embedded in the substrate, and act as displacement markers to trace substrate deformation. Typical substrates used for TFM include transparent hydrogels such as poly acrylamide (PAA), polyethylene glycol PEG), polydimethylsiloxane silicone (PDMS), and silicon-based gels [25]. All these substrates are in the linear response regime, they have isotropic properties in response to external forces and exhibit significant deformations, thus enabling very accurate, high-resolution measurements of the traction forces.

**Figure 2:**
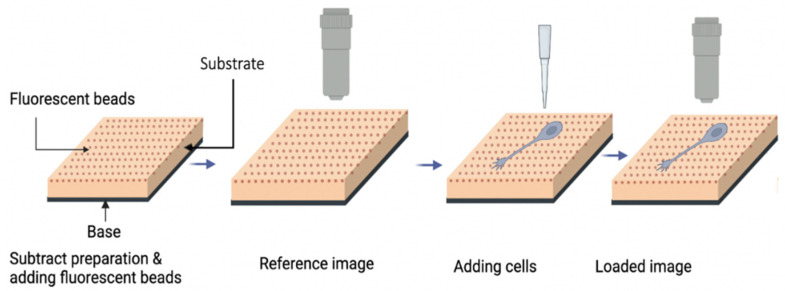
Schematic of the basic procedures used in traction force microscopy (detailed description given in the main text).

To measure the substrate deformation, two images (loaded and reference) are taken using the fluorescent microscope (Figure 2). The loaded image is taken when the cells exert traction forces on the substrate and the reference image is taken when the substrate is in a relaxed state, without the traction forces from the cells. By comparing these two images, the displacement field of the deformed substrate can be measured using image processing software.

**Figure 3:**
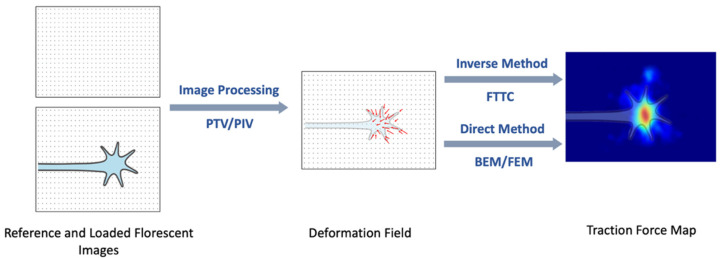
Schematic description of how traction forces are reconstructed from the measured substrate displacement (deformation field). The figure shows the schematic reconstruction for a growth cone, which leads the extension of an axon. The bright red area on the right image corresponds to the region where the growth cone exerts high traction forces.

Several types of image processing methods, including particle tracking/image velocimetry (PTV/PIV), correlation-based PTV [32], and feature-based registration [33], are typically used to determine the displacement field. After obtaining the displacement field the traction force can be calculated using a variety of computational methods [23,26,27,28,29,30,31,32,33,34,35,36,37,38,39]. Since the cells exert distributions of forces rather than discrete traction forces, the TFM image is interpreted as traction stress, i.e., force per unit area. The actual traction force can be estimated by integrated the stress over a given contact area [27,28,29,30,31,32,33,34,35]. The computational approaches can be divided into the direct and inverse methods (Figure 3). In the direct method, the traction force is calculated directly from the displacement field by differentiation and transformation between strain and stress. Finite element methods (FEM) [33,34] and boundary element calculations (BEM) [32,35] are the two most used direct methods for calculating the traction force. The inverse method minimizes the difference between the experimental and estimated displacement fields. Fourier Transform Traction Cytometry (FTTC) is the most commonly used inverse method for the reconstruction of traction forces [28,32,33,34,35]. FTTC was introduced by Butler et al. [32] and is based on modeling the deformation field using the linear theory of elasticity, which leads to a Fredholm-type integral equation [32,36]. In FTTC the spatial displacement field is discretized using a uniform rectangular grid, that covers the whole image. The traction force field is then calculated as a set of plane waves using the Boussinesq Green function [32,33,34,35].

In this work, we calculate the traction stress field from the deformation field by using an FTTC algorithm as presented in references [34,35,39]. This method relies on the general relationship between the applied force on a solid object and the resulting deformations, which is valid in the linear elastic regime [36]. We are briefly summarizing this method below.

Consider any point with its position vector before the deformation $\vec{r}$ (with components *x_i_ = x*, *x_j_ = y* and *x_k_ = z*), and after the deformation due to traction force ${\vec{r}}^{\prime }$, respectively. Then, the deformation of this point due to the external forces is given by the vector |$\vec{r}-{\vec{r}}^{\prime }$|. The displacement vector $\vec{u}\;$of the substrate is then given by:

(1) $$\vec{u}={\vec{r}}^{\prime }-\vec{r}$$

For small deformations, the general equation that relates the displacements $\vec{u}$ with the traction field $\vec{T}$ can be written as a convolution (Boussinesq approximation):

(2) $$\vec{u}=G\left(r\right)\vec{T}\left(r\right)$$

where *G* is the Green’s function of the problem, i.e., the Fredholm integral for TFM [32,33,34,39]. To find $\vec{T}$, the displacement field $\vec{u}$ is interpolated onto a rectangular grid where the spatial variable is discretized, and the traction force is described by a set of plane waves. The wave vectors $\vec{k}$ of the induced uniform grid in Fourier space is determined by the sampling grid. By taking the inverse Fourier transform of Equation (2), we obtain the solution for the traction field:

(3) $$\vec{T}={\mathrm{FT}}_{2}^{-1}\left({\tilde{G}}^{-1}\tilde{\vec{u}}\right)$$

where ${\mathrm{FT}}_{2}^{-1}$ denotes the (two-dimensional) inverse Fourier transform operation, and the symbol ~ denotes the Fourier transform for the function with wave vector $\vec{k}$. The expression for $\tilde{G}$ can be written as [32,33,34,39]:

(4) $$\tilde{G}\left(\vec{k}\right)=A\frac{2\pi }{k^{3}}\left[\begin{array}{cc} \left(1-\sigma \right)k^{2}+\sigma k_{y}^{2} & \sigma k_{x}k_{y}\\ \sigma k_{x}k_{y} & \left(1-\sigma \right)k^{2}+\sigma k_{x}^{2} \end{array}\right]$$

where *A* = $\frac{\left(1+\sigma \right)}{\pi E},\;\mathrm{with}\;\sigma$ and *E* being the Poisson’s ratio and the Young’s modulus of the substrate, respectively. Equations (1)–(4) relate the traction forces with the corresponding substrate deformations.

TFM initially emerged as a powerful microscopic technique for measuring the force generated by a single cell in two dimensions (2D). However, it was developed over time to include multicellular systems and 3-dimensional (3D) substrates. TFM is also used to measure cellular health since the movement of the cells is primarily governed by biomechanical interactions [25,26]. A growing body of research explores mechanobiological and cellular mechano-transduction mechanisms by studying the interaction between the cells and ECM through traction force. For example, traction forces exerted by 3T3 fibroblasts during steady locomotion was mapped by Dembo and Wang in reference [37]. The authors found that the propulsive thrust for fibroblast locomotion is around 200 nN [37]. Razafiarison et al. [38] showed that surface energy of soft biomaterials can affect the receptor recruitment and signaling of human mesenchymal stem cell cells by using TFM. It was found that the traction forces generated by the different types of cancer cells are different from each other and they can be distinguished from the normal cells. Thus, TFM has become a very important characterization technique for identifying various types of cancer cells, as summarized in a recent paper by Zancla et al. [39].

Compared to other types of cells, there are relatively few papers in the literature that have used TFM to study neuronal growth and axonal dynamics [28,29,30,31]. In an important work, Koch and collaborators used TFM to compare the biomechanical properties of Peripheral (DRG) and Central Nervous System (CNS) hippocampal neurons [28]. The study found that the traction force increased with increasing substrate stiffness for both type of neurons. However, the growth cones of the DRG neurons exerted forces that were several times larger compared to the CNS neurons. DRG axons displayed maximum outgrowth on the substrate, with a Young’s modulus of around 1000 Pa. In contrast, the outgrowth for axons of CNS neurons was independent of the substrate stiffness [28].

In a different report, Polackwich et al. measured the dynamics of traction stresses of growth cones of postnatal Dorsal Root Ganglia (DRG) neurons obtained from the lumbar region of P0-P1 rat pups [29]. This paper reported that the stress field distribution within the growth cone fluctuated over time, with the strongest average stress field measured around the center of the growth cone and directed towards the neck of the growth cone. To study the localized stress peak lifetime, high-time-resolution measurements were performed, with an image captured every 2 s. Hyland and collaborators have used TFM to measure traction forces exerted by Aplysia bag cell neurons [30]. The authors found that the growth cones generated traction force in the peripheral domain that was pointed away from the leading edge and toward the central domain (see Figure 1a and Section 4). They also reported that the tension was stable during outgrowth, while the traction patterns were extremely dynamic. The total force integrated over the entire growth cone was found to be in the range 1–20 nN [30]. In [31], Franze and collaborators measured the mechanical deformations and stresses exerted by growth cones on substrates with known elastic modulus and showed that the mechanical properties of the substrate acted as guidance cues for axonal growth.

## 3. Results

### 3.1. Results on Combined Atomic Force and Fluorescence Microscopy Measurements of Neurons

**Figure 4:**
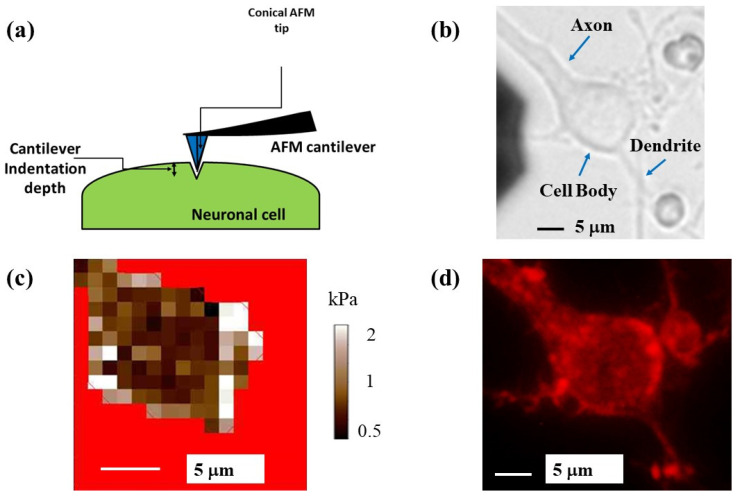
(**a**) Schematic of the AFM indentation experiments for a conical tip. The cell body is represented as an elongated shape (highlighted in green). The figure also shows the deformation of the cell soma, which is equal to the indentation depth of the AFM tip; (**b**) bright-field optical image of a neuron showing the main structural components of the cell; (**c**) elasticity map for the cell shown in (**b**), acquired with a cantilever with spring constant of 0.03 N/m; (**d**) fluorescence image of the same neuron stained for actin. Regions of high actin density correspond to the bright red areas. The cell body regions that display high concentrations of actin correlate with higher-than-average values of the elastic modulus shown in (**c**).

**Figure 5:**
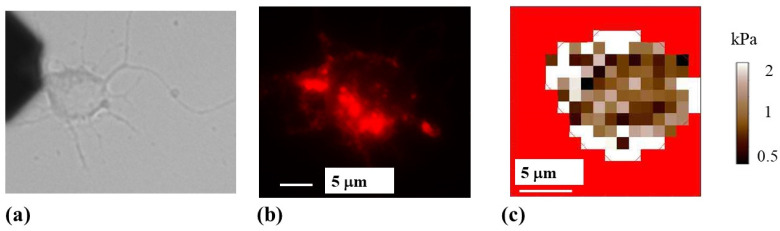
(**a**) Bright-field optical image of a neuron. The AFM cantilever appears on the left side of the image; (**b**) fluorescence image of the same neuron stained for actin. Regions of high actin density correspond to the bright red areas; (**c**) elasticity map for the cell shown in (**a**,**b**). The elasticity map was acquired with a cantilever with spring constant of 2.8 N/m. The cell body regions that display higher-than-average values of the elastic modulus correlate with regions with high concentration of actin. The location of regions with high actin concentration does not change significantly during the AFM measurements.

By performing simultaneous AFM and FM experiments, we acquire elasticity maps (measurements of the elastic modulus at different points on the cell) and correlate these maps with intracellular components and cytoskeletal rearrangements for individual neuronal cells. Figure 4 shows an example of this type of experiment for a single cortical neuron. Figure 4a shows the schematic for the AFM force–indentation experiment: the conical AFM tip compresses the neuron at different locations along the cell. Figure 4b shows a bright-field optical image of a neuron. The main structural components—axon, cell body (soma), and dendrites—are also indicated in the figure. Figure 4c displays the AFM-acquired elasticity map for the soma of the same cell. The measured values of the elastic modulus range from 0.4 to 2.3 kPa. Figure 4d displays a fluorescent image of the cell stained for actin. The regions of high actin concentration correspond to the regions of high fluorescence intensity (bright red), mainly along the top and right-hand side of the cell, as well as close to the axon junction. Similar bright-field and fluorescence images, as well as AFM elasticity maps for three additional neuronal cells, are shown in Figure 5 and in the Supplementary Materials (Figures S1 and S2).

Figure 4c,d, and Figure 5b,c (as well as Figures S1 and S2 in the Supplementary Materials) show a high degree of overlap between the regions of the cell displaying high actin concentration and the regions with high values of elastic modulus *E* ≥ 1 kPa, corresponding to bright colored areas of the elasticity map. The degree of overlap between regions of the soma with higher-than-average elastic modulus (*E* ≥ 1 kPa) and regions with high actin concentration for these cells is in the range 78–85%. We note that the location of the regions with high actin concentration does not change significantly inside the cell during the AFM measurements. Moreover, we measure similar values for the elastic moduli and similar elasticity maps for different cells, regardless of whether soft (0.03 N/m, Figure 4 and Figure S1) or stiffer (2.8 N/m, Figure 5 and Figure S2) cantilevers are used. These results are in agreement with our previous reports, in which we demonstrated that the regions of the cell soma with *E* ≥ 1 kPa were associated with the main components of the cytoskeleton [12,13,14].

### 3.2. Results on Combined Traction Force, Atomic Force, and Fluorescence Microscopy Measurements of Cortical Neurons

**Figure 6:**
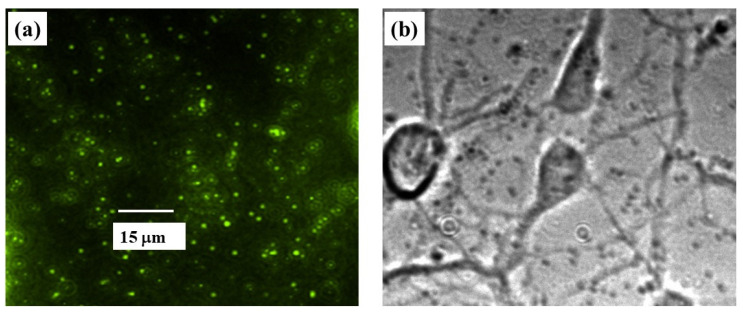
(**a**) Example of reference fluorescent image of the PAA substrate with embedded FluoSphere beads; (**b**) optical bright-field image of cortical neurons cultured on the substrate shown in (**a**). The scale bar shown in (**a**) is the same for both images.

Here we use the inverted stage of the MFP3D Asylum Research AFM to implement TFM using this instrument, and thus to perform simultaneous combined AFM/TFM/FM measurements. In this way, changes in elastic modulus and cell deformations can be directly related to forces/stresses exerted by the cell on the surface. Furthermore, by combining TFM and FM we identify cell–substrate adhesion points and correlate these points with traction forces exerted by the cell. Cortical neurons are cultured on poly D-lysine-coated PAA hydrogels with embedded fluorescent FluoSphere beads (see Section 2.1). The deformation of the PAA substrate is determined by tracking the fluorescent beads embedded into the substrate, as described in Section 2.3. For each measurement, we record a set of fluorescence images of beads (Figure 6a), as well as bright-field transmission images of cells (Figure 6b).

The position of the beads of loaded and reference images is recorded using the Nikon Eclipse Ti inverted stage microscope integrated with the AFM, the deformation is measured by comparing the bead positions in the two images, and the traction stress is calculated as described in Section 2.3.

**Figure 7:**
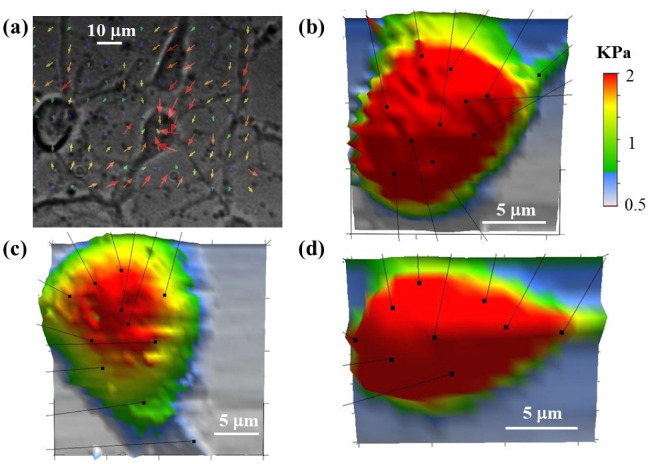
(**a**) Traction Force Microscopy image for three cortical neurons cultured on a PAA substrate with Young’s modulus *E_S_* = 470 Pa. The arrows show the displacement field $\vec{u}$. The size of the arrow at each point is equal to the magnitude of the displacement at that point; (**b**–**d**) examples of 3D elasticity maps measured by AFM for three neuronal cells. The arrows in each figure show the points of maximum coupling between the cell and the growth substrate. The stiffness scale bar shown in (**b**) is the same for all AFM images.

In addition to TFM experiments, we perform simultaneous high-resolution AFM measurements of individual neuronal cells. Figure 7 shows an example of these combined TFM/AFM measurements. Figure 7a shows a TFM image of neuronal cells cultured on a PAA substrate with Young’s modulus *E_S_* = 470 Pa. The arrows show the displacement field $\vec{u}$. Figure 7b–d show three-dimensional (3D) AFM elasticity maps of single neurons. The AFM measurements enable us to determine the distribution of elastic modulus, cell morphology and volume. In this way changes in cell stiffness and cell deformations measured by AFM can be directly related to forces exerted by the cell on the growth surface, which are simultaneously measured by TFM. The arrows in Figure 7b–d indicate the position of focal adhesion points of the cell. The AFM data (Figure 7b–d) demonstrate that neurons that exert traction forces on the substrate have higher-than-average values of the cellular elastic modulus, i.e., in the range 1–2 kPa.

**Figure 8:**
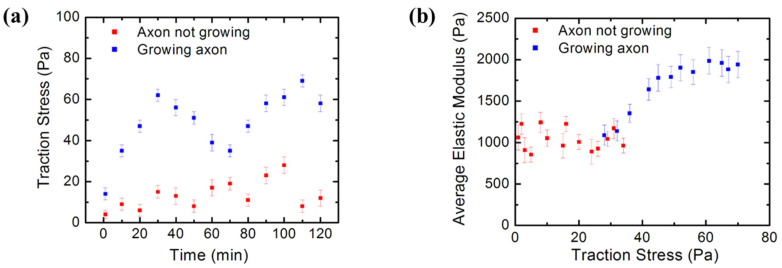
(**a**) Traction Force Microscopy data showing the time evolution of the traction stress for two different cells: a neuron with a growing axon (blue data points), and a different neuron with no active growing processes (red data points). The blue data points show that the growth dynamics is characterized by intermittent growth phases of relatively high traction stress, alternating with periods of low stress; (**b**) simultaneous AFM-TFM measurements of the variation of the average neuron elastic modulus (measured by AFM) with the traction stress exerted by the cell on the growth substrate (measured by TFM). Blue points show data measured for neurons with axons in the active growth phase. Red data points show measurements performed on neurons that are not undergoing active growth processes. Error bars in both figures represent the standard error of the mean. Both data series were acquired at *t* = 32 h after cell plating. The figure demonstrates that cortical neurons stiffen as the axons extend.

Traction stresses and forces are determined from the measured bead displacement on the PAA substrate using Equations (1)–(4) (Section 2.3) and a custom MATLAB routine [34]. Figure 8a shows an example of the variation of the traction stress in time for two different cells: a cell with an active growing axon (blue data points), and a cell where the axon is not growing (red data points). The measurements were performed every 10 min for a total of 2 h, starting at *t* = 32 h after cell culture.

The blue data points in Figure 8a demonstrate that growing cortical neurons are highly dynamic, displaying periods of relatively high traction stress, which alternate with periods of low stress. In contrast, the stress generated by neurons that do not display active growth (red data points) is relatively constant, exhibiting lower overall stress values than the growing cells. Figure 8b shows the variation of the average neuron elastic modulus (measured by AFM) with the traction stress exerted by the cell on the growth substrate (measured by TFM). These combined AFM–TFM measurements were performed on neurons with growing axons (blue data points), as well as on neurons without active processes (red data points). These experiments clearly demonstrate that the cortical neurons stiffen as the axons extend. The ability of cortical neurons to change their elastic modulus is very important for regulating axonal growth on the mechanically inhomogeneous environments found in the brain [9,10].

**Figure 9:**
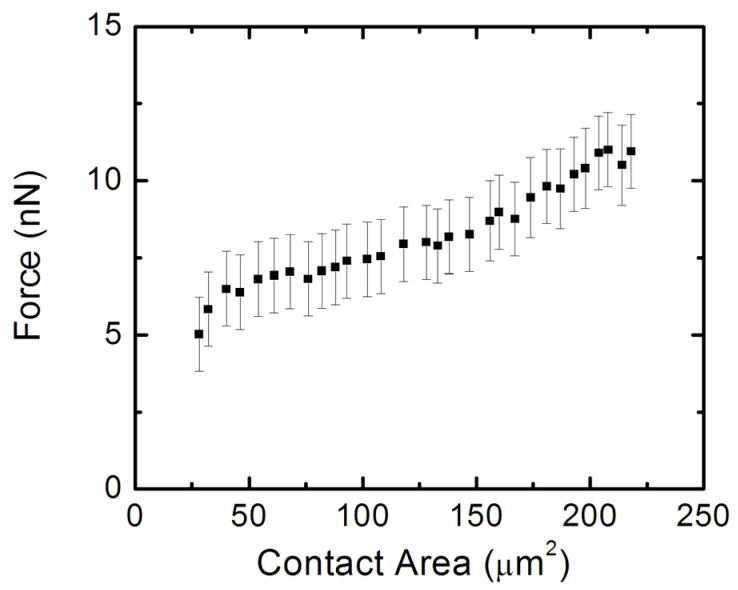
Plot of total traction force against the contact area of the cell for several neurons with growing axons. Error bars represent the standard error of the mean. The traction force increases with increasing contact area, as predicted by the contact guidance model.

Figure 9 shows the variation in the traction force (obtained from TFM) with the cell–surface contact area (measured by AFM). These measurements are performed for several neurons (*N* = 30 cells) that display axonal growth. The data in Figure 9 show a net increase in the traction force with increasing contact area, consistent with a contact guidance mechanism for axonal growth. Neurons generate traction forces as they extend, and axonal growth proceeds by the formation of adhesion points between the cell and the substrate.

To our knowledge, these measurements represent the first report in the literature on the traction forces applied by the cortical neurons on the growth substrate, as well as the first report of the variation of neuron elastic modulus with traction stresses exerted by the cell. These results are in agreement with our previous results, which showed an increase in the stiffness of the neurons during axonal growth [12]. However, in our previous work [12] we observed axonal growth using optical microscopy and did not measure the actual traction stresses and forces applied by the cell during growth. We also demonstrated that the cell stiffening during axonal growth was due to changes in cytoskeletal dynamics, which result in the redistribution of microtubules and actin filaments during axonal extension [12]. These cytoskeletal changes, as well as the TFM and AFM data are consistent with a contact guidance mechanism for neuronal growth, as discussed below.

## 4. Discussion

Neurons respond to a variety of external cues during axonal outgrowth, and as a result, the growth cone generates traction forces that guide the axon. In previous work, we investigated axonal growth on micropatterned surfaces, and showed that axons follow external cues through a contact guidance mechanism, in which the growth cone detects environmental stimuli and adjusts its motion in response to geometrical and mechanical features present on the growth substrate [20,21,22].

In this paper, we performed TFM experiments to measure the traction stresses and forces exerted by cortical neurons on the PAA growth substrates. Simultaneous AFM experiments demonstrated an increase in the elastic modulus of the cell during axonal extension. The AFM data (Figure 7b–d and Figure 8b) demonstrated that neurons that exert traction forces on the substrate have higher-than-average values of cellular elastic modulus, in the range 1–2 kPa. The TFM data (Figure 7a and Figure 8) showed the measured traction stresses exerted by the growing cortical neurons to be in the range 28–70 Pa, corresponding to traction forces in the range 5–11 nN (Figure 9). In contrast, neurons without active growing processes displayed traction stresses in the range 0–34 Pa (Figure 8). These values were larger by a factor 3 to 5 than the corresponding values reported for hippocampal neurons on substrates of similar stiffness, and close to the peak values obtained for peripheral DRG neurons [28]. The traction forces measured in our experiments are smaller than the average values measured for Aplysia bag neurons [30].

These results demonstrate that neuronal growth is governed by a contact guidance mechanism. Contact guidance is the ability of cells to adjust their motion in response to external cues present in the surrounding environment. This behavior has been observed for many types of cells including neurons, fibroblasts, and cancerous cells [19,20,21,22,40,41,42,43,44]. However, despite its important role in cellular motility, little is known about the intracellular mechanisms that regulate contact guidance. It is generally accepted that, for contact guidance to work, the exterior extracellular matrix (ECM) substrate has to interact with the intracellular actin filaments. This interaction is mediated by a special type of protein called integrins and focal adhesion points that connect the cell with the substrate [40,41,42,43,44]. Integrin-based complexes and focal adhesions (FAs) on the edges of lamellipodia attract and adhere actin to the ECM. Previous work has shown that contact guidance requires the activation of specific chemical signaling pathways [41,42,43,44]. In particular, Rho-associated kinase (ROCK), a protein that mediates the transfer of a phosphate group to a specific molecule, increases myosin-II contractility, and promotes the formation of FAs [41,42,43,44]. For example, Riveline and collaborators found two phases of focal adhesion formation [43]. The first, involving contractile force, requires total ROCK activation. The second, which is the development of focal adhesion response, requires only mDia1 (a downstream formin homology protein), which interacts with profilin to increase actin activity. Other chemical signaling contributions to contact guidance include the activation of the Rho-family g-protein, which is involved in the regulation of ECM adhesion and the formation of focal adhesions [43,44]. Regardless of the exact details of the intercellular mechanisms, the main signature of contact guidance is an increase in the traction force with increasing cell–substrate contact area [40,41]. Figure 9 demonstrates that this is indeed the case for cortical neurons that have growing axons.

**Figure 10:**
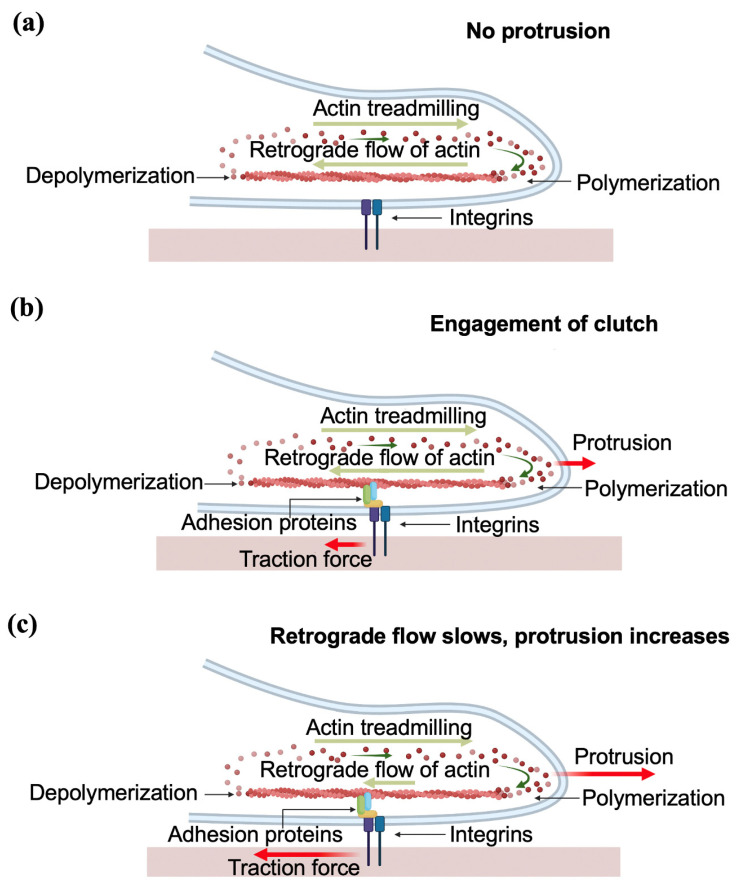
Schematic representation of axonal outgrowth, showing the stage of protrusion. (**a**) When the molecular clutch is not engaged, the F-actin treadmilling and retrograde flow is balanced, and there is no traction or protrusion; (**b**) the clutch is engaged through adhesion proteins; (**c**) the engagement of the clutch results in the attenuation of retrograde actin flow, and in an increase of F-actin polymerization at the leading edge, which causes the generation of traction forces and the protrusion of the growth cone.

In the case of neurons, the contact guidance mechanism has several specific features. Generation of traction force occurs when the growth cone adheres to the substrate through integrin-containing FAs which act as a mechanical “clutch”. This mechanism, first proposed by Mitchison and Kirschner in 1988, was described as the “molecular clutch” hypothesis, and it is also called the “substrate–cytoskeletal” model [45]. More specifically, within this model, the outgrowth of the axon occurs through three stages: protrusion, engorgement, and consolidation. In the first stage, the contact between the growth cone and the substrate leads to the formation of a “molecular clutch”, which anchors the cytoskeleton to the substrate and enables the growth cone to proceed with the protrusion. During the protrusion, with the assistance of the clutch, the filopodia and lamellipodia of the peripheral (P)-domain move forward (Figure 10). Next, during the engorgement stage, the central (C)-domain (Figure 1) moves forwards to the site of new growth. Finally, in the consolidation stage, the formation of a new segment of axon shaft occurs in the recently advanced central (C)-domain [2].

More specifically, the clutch model explains the protrusion of the growth cone, and depends on the actin dynamics within the cytoskeleton [2,45]. The protrusion is primarily governed by two processes: F-actin treadmilling and the actin retrograde flow (Figure 10). F-actin treadmilling consists of the polymerization of actin at the leading end of the P-domain, which results in the membrane being pushed forward. The retrograde flow is the backward flow of F-actin (i.e., flow oriented towards the center of the growth cone, or the T-zone, in Figure 1), and leads to the recycling of the actin in the filopodia.

The molecular clutch connects the flow of F-actin and the integrins of the ECM. During protrusion, the F-actin adheres to the substrate through focal adhesions (clutch molecules), which in turn causes the attenuation of the retrograde flow of F-actin (Figure 10b). The momentum of F-actin retrograde flow is transmitted to the substrate through the clutch, thus generating the traction force on the substrate. At the same time, the F-actin treadmilling at the leading end pushes the membrane forwards, resulting in the advancement of the growth cone through a Brownian ratchet mechanism [46,47] (Figure 10c). The whole process is controlled by the contractility of the motor protein myosin II [46].

In summary, the clutch model shows that traction stresses emerge from coupling between cell cytoskeleton and the growth substrate. Thus, the clutch model predicts an increase in the traction stress during the phases of active axonal growth (as shown Figure 8a), as well as an increase in cytoskeletal stiffness with increasing traction stress (as shown in Figure 8b). When the clutch is engaged, the interactions between actin filaments, myosin II and focal adhesions increase the mechanical tension in the axon. This results in the overall stiffening of the cell, as shown by simultaneous AFM/TFM measurements (Figure 7 and Figure 8b). In this case, the cytoskeletal components act as stiff load-bearing structures that generate surface adhesion and traction forces. Furthermore, the contraction of myosin II causes the disengagement of the clutch, which results in a decrease in the traction force exerted on the substrate [2]. In this case, the two processes, treadmilling and retrograde flow, are balanced, the protrusion does not occur, and the growth cone does not advance [2]. Therefore, the clutch model predicts that for neurons where the axons do not extend, decreases in the traction stresses and in the overall value of the cell elastic modulus should be observed. This is indeed confirmed by our AFM measurements (red data points in Figure 8).

One of the key questions in neuronal growth is: how do the observed growth dynamics emerge from cell–cell and cell–substrate interactions? To address this question, in future work, we will perform combined TFM/FM/AFM experiments to quantify the biomechanical interactions between neurons and their growth environment. Specifically, we will create in vitro neuronal circuits grown on tunable biomaterial substrates that mimic the environments that the cells encounter in vivo. We will use these techniques to measure and quantify: (a) the biomechanical properties of neuronal cells (elastic moduli, axonal tension, cell volume and morphology); (b) cell cytoskeletal dynamics (polymerization of microtubules and actin filaments, generation of traction forces between the cell and the growth substrate); (c) axonal dynamics and the mechanisms of network formation through contact guidance and translation of mechanical input into intracellular response. In particular, we will investigate how changes in internal cellular processes, such as changes in the expression of ROCK and BAR proteins, or the opening of stress-activated ion channels, affect the generation of traction forces and the biomechanical properties of neurons. These experiments will allow us to elucidate the collaborative mechanisms among the many biochemical and biomechanical factors that control neuronal growth and function.

In principle, these future investigations will enable researchers to quantify the influence of environmental cues (geometrical, mechanical, biochemical) on cellular dynamics, and to relate the observed cell motility behavior to cellular processes, such as cytoskeletal dynamics, cell–surface interactions, and signal transduction.

## 5. Conclusions

Although it is generally accepted that mechanics plays an important role in neuronal growth and axonal dynamics, this field is still underdeveloped compared to other areas of neuroscience, and we are only beginning to understand how biomechanical processes affect the development of neuronal circuits and the function of neurons. To make significant progress in this field, we need new high-resolution imaging techniques. In this paper, we present the first, to our knowledge, measurements of the biomechanical properties of individual neurons and of the axon-substrate traction forces using combined atomic force, traction force, and fluorescence microscopy. We report high-resolution measurements of neuron elastic moduli and relate these values to traction forces exerted by the cells on the growth substrate. Our results show that neuronal growth is regulated by a contact guidance mechanism, in which the axon responds to external mechanical cues. This combination of powerful experimental techniques can also be applied to other types of cells to give new insights into the nature of cellular dynamics. This will enable researchers to measure cell–cell and cell–environment interactions, and to quantify the influence of environmental cues on cellular dynamics and cellular biomechanical properties.

## Data Availability

The data presented in this study are available within the manuscript and its Supplementary Materials.

## Supplementary Materials

The following supporting information can be downloaded at: https://www.mdpi.com/article/10.3390/biomimetics7040157/s1, Figures S1 and S2: Additional examples of bright field, fluorescence and elasticity maps of neurons.

## References

[1] Huber A.B., Kolodkin A.L., Ginty D.D., Cloutier J.F. (2003). Signaling at the growth cone: Ligand-receptor complexes and the control of axon growth and guidance. Annu. Rev. Neurosci..

[2] Lowery L.A., Vactor D.V. (2009). The trip of the tip: Understanding the growth cone machinery. Nat. Rev. Mol. Cell Biol..

[3] Wen Z., Zheng J.Q. (2006). Directional guidance of nerve growth cones. Curr. Opin. Neurobiol..

[4] Tessier-Lavigne M., Goodman C.S. (1996). The molecular biology of axon guidance. Science.

[5] Dickson B.J. (2002). Molecular mechanisms of axon guidance. Science.

[6] Staii C., Viesselmann C., Ballweg J., Shi L., Liu G.-Y., Williams J.C., Dent E.W., Coppersmith S.N., Eriksson M.A. (2011). Distance dependence of neuronal growth on nanopatterned gold surfaces. Langmuir.

[7] Rosoff W.J., Urbach J.S., Esrick M.A., McAllister R.G., Richards L.J., Goodhill G.J. (2004). A new chemotaxis assay shows the extreme sensitivity of axons to molecular gradients. Nat. Neurosci..

[8] Staii C., Viesselmann C., Ballweg J., Shi L., Liu G.-Y., Williams J.C., Dent E.W., Coppersmith S.N., Eriksson M.A. (2009). Positioning and guidance of neurons on gold surfaces by directed assembly of proteins using atomic force microscopy. Biomaterials.

[9] Franze K., Guck J. (2010). The biophysics of neuronal growth. Rep. Prog. Phys..

[10] Franze K. (2013). The mechanical control of nervous system development. Development.

[11] Lamoureaux P., Ruthel G., Buxbaum R.E., Heidemann R.E. (2002). Mechanical tension can specify axonal fate in hippocampal neurons. J. Cell Biol..

[12] Spedden E., White J.D., Naumova E.N., Kaplan D.L., Staii C. (2012). Elasticity maps of living neurons measured by combined fluorescence and atomic force microscopy. Biophys. J..

[13] Spedden E., Kaplan D.L., Staii C. (2013). Temperature response of the neuronal cytoskeleton mapped via atomic force and fluorescence microscopy. Phys. Biol..

[14] Sunnerberg J.P., Moore P., Spedden E., Kaplan D.L., Staii C. (2019). Variations of elastic modulus and cell volume with temperature for cortical neurons. Langmuir.

[15] Spedden E., Wiens M.R., Demirel M.C., Staii C. (2014). Effects of surface asymmetry on neuronal growth. PLoS ONE.

[16] Beighley R., Spedden E., Sekeroglu K., Atherton T., Demirel M.C., Staii C. (2012). Neuronal alignment on asymmetric textured surfaces. Appl. Phys. Lett..

[17] Yurchenko I., Vensi Basso J.M., Syrotenko V.S., Staii C. (2019). Anomalous diffusion for neuronal growth on surfaces with controlled geometries. PLoS ONE.

[18] Rizzo D.J., White J.D., Spedden E., Wiens M.R., Kaplan D.L., Atherton T.J., Staii C. (2013). Neuronal growth as diffusion in an effective potential. Phys. Rev. E.

[19] Vensi Basso J.M., Yurchenko I., Simon M., Rizzo D.J., Staii C. (2019). Role of geometrical cues in neuronal growth. Phys. Rev. E.

[20] Yurchenko I., Farwell M., Brady D.D., Staii C. (2021). Neuronal growth and formation of neuron networks on directional surfaces. Biomimetics.

[21] Sunnerberg J.P., Descoteaux M., Kaplan D.L., Staii C. (2021). Axonal growth on surfaces with periodic geometrical patterns. PLoS ONE.

[22] Descoteaux M., Sunnerberg J.P., Brady D.D., Staii C. (2022). Feedback-controlled dynamics of neuronal cells on directional surfaces. Biophys. J..

[23] Hur S.S., Jeong J.H., Ban M.J., Park J.H., Yoon J.K., Hwang Y. (2020). Traction force microscopy for understanding cellular mechanotransduction. BMB Rep..

[24] Harris A.K., Wild P., Stopak D. (1980). Silicone Rubber Substrata: A new wrinkle in the study of cell locomotion. Science.

[25] Roca-Cusachs P., Conte V., Trepat X. (2017). Quantifying forces in cell biology. Nat. Cell Biol..

[26] Style R.W., Boltyanskiy R., German G.K., Hyland C., MacMinn C.W., Mertz A.F., Wilen L.A., Xu Y., Dufresne E.R. (2014). Traction force microscopy in physics and biology. Soft Matter.

[27] Lemmon C.A., Romer L.H. (2010). A predictive model of cell traction forces based on cell geometry. Biophys. J..

[28] Koch D., Rosoff W.J., Jiang J., Geller H.M., Urbach J.S. (2012). Strength in the periphery: Growth cone biomechanics and substrate rigidity response in peripheral and central nervous system neurons. Biophys. J..

[29] Polackwich R.J., Koch D., McAllister R., Geller H.M., Urbach J.S. (2015). Traction force and tension fluctuations in growing axons. Front. Cell. Neurosci..

[30] Hyland C., Mertz A.F., Forscher P., Dufresne E. (2014). Dynamic peripheral traction forces balance stable neurite tension in regenerating Aplysia bag cell neurons. Sci. Rep..

[31] Franze K., Gerdelmann J., Weick M., Betz T., Pawlizak S., Lakadamyali M., Bayer J., Rillich K., Gögler M., Lu Y.B. (2009). Neurite branch retraction is caused by a threshold-dependent mechanical impact. Biophys. J..

[32] Butler J.P., Tolić-Nørrelykke I.M., Fabry B., Fredberg J.J. (2002). Traction fields, moments, and strain energy that cells exert on their surroundings. Am. J. Physiol. Cell Physiol..

[33] Yang Z., Lin J.S., Chen J., Wang J.H. (2006). Determining substrate displacement and cell traction fields—A new approach. J. Biol.

[34] Han S.J., Danuser G., Fabry B. Matlab Routines Traction Force Microscopy, Public Repository. https://github.com/topics/traction-force-microscopy.

[35] Sabass B., Gardel M.L., Waterman C.M., Schwarz U.S. (2008). High resolution traction force microscopy based on experimental and computational advances. Biophys. J..

[36] Landau L., Lifshitz E.M. (1986). Theory of Elasticity.

[37] Dembo M., Wang Y.-L. (1999). Stresses at the cell-to-substrate interface during locomotion of fibroblasts. Biophys. J..

[38] Razafiarison T., Holenstein C.N., Stauber T., Jovic M., Vertudes E., Loparic M., Kawecki M., Bernard L., Silvan U., Snedeker J.G. (2018). Biomaterial surface energy-driven ligand assembly strongly regulates stem cell mechanosensitivity and fate on very soft substrates. Proc. Natl. Acad. Sci. USA.

[39] Zancla A., Mozetic P., Orsini M., Forte G., Rainer A. (2022). A primer to traction force microscopy. J. Biol. Chem..

[40] Moore S.W., Sheetz M.P. (2011). Biophysics of substrate interaction: Influence on neural motility, differentiation, and repair. Dev. Neurobiol..

[41] Ray A., Lee O., Win Z., Edwards R.M., Alford P.W., Kim D., Provenzano P.P. (2017). Anisotropic forces from spatially constrained focal adhesions mediate contact guidance directed cell migration. Nat. Commun..

[42] Mammoto A., Mammoto T., Ingber D.E. (2012). Mechanosensitive mechanisms in transcriptional regulation. J. Cell Sci..

[43] Riveline D., Zamir E., Balaban N.Q., Schwarz U.S., Ishizaki T., Narumiya S., Kam Z., Geiger B., Bershadsky A.D. (2001). Focal contacts as mechanosensors: Externally applied local mechanical force induces growth of focal contacts by an mDia1-dependent and ROCK-independent mechanism. J. Cell Biol..

[44] Buskermolen A.B.C., Ristori T., Mostert D., Van Turnhout M.C., Shishvan S.S., Loerakker S., Kurniawan N.A., Deshpande V.S., Bouten C.V.C. (2020). Cellular contact guidance emerges from gap avoidance. Cell Rep. Phys. Sci..

[45] Mitchison T., Kirschner M. (1988). Cytoskeletal dynamics and nerve growth. Neuron.

[46] Medeiros N.A., Burnette D.T., Forscher P. (2006). Myosin II functions in actin-bundle turnover in neuronal growth cones. Nat. Cell Biol..

[47] Démoulin D., Carlier M.-F., Bibette J., Baudry J. (2014). Power transduction of actin filaments ratcheting in vitro against a load. Proc. Natl. Acad. Sci. USA.

