# Supplementary material for: Combined Traction Force–Atomic Force Microscopy Measurements of Neuronal Cells
Source: Biomimetics (Basel). 2022 Oct 8;7(4):157. doi: 10.3390/biomimetics7040157 (PMC9624305; doi:10.3390/biomimetics7040157)
Supplement: Supplementary file 1 [file biomimetics-07-00157-s001.zip › biomimetics-1925693-supplementary.pdf]

Supplemental Material for

Combined traction force – atomic force microscopy measurements of neuronal cells

Udathari Kumarasinghe [1], Lucian N. Fox[1], Cristian Staii[1,*]

1. Department of Physics and Astronomy, Tufts University, Medford, Massachusetts 02155, USA

[*] Corresponding Author: Prof. C. Staii, E-mail: Cristian.Staii@tufts.edu

Keywords: Neuron, Axonal Growth, Traction Force Microscopy, Atomic Force Microscopy, Cellular Mechanics, Tissue Engineering

Additional Experimental Data

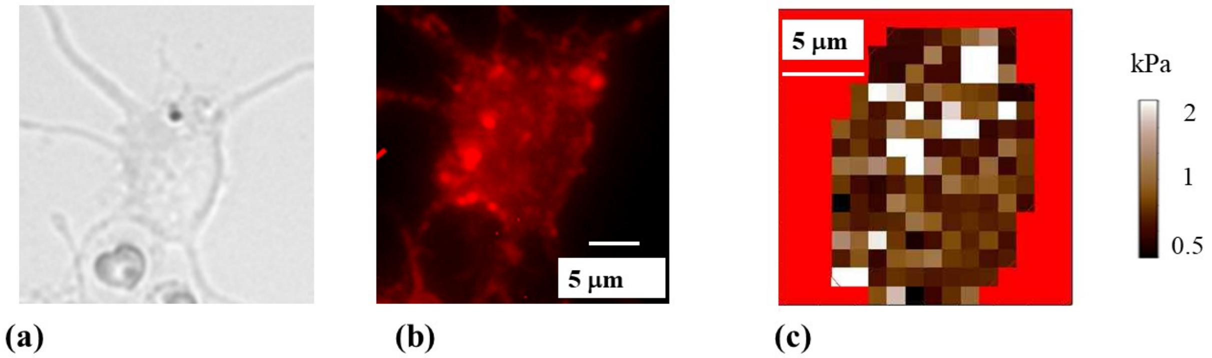

Figure S1. (**a**) Bright field optical image of a neuron. (**b**) Fluorescence image of the same neuron stained for actin. Regions of high actin density correspond to the bright red areas. (**c**) Elasticity map for the cell shown in (**a**) and (**b**). The elasticity map was acquired with a cantilever with spring constant of 0.03 N/m. The cell body regions that display higher - than - average values of the elastic modulus correlate with regions with high concentration of actin. The location of the regions with high actin concentration does not change significantly during the AFM measurements.

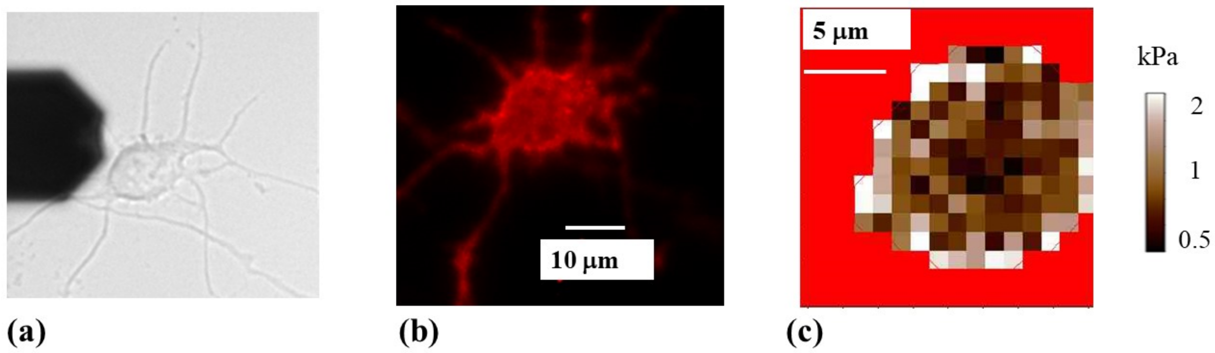

Figure S2. (**a**) Bright field optical image of a neuron. The AFM cantilever appears on the left side of the image. (**b**) Fluorescence image of the same neuron stained for actin. Regions of high actin density correspond to the bright red areas. (**c**) Elasticity map for the cell shown in (**a**) and (**b**). The elasticity map was acquired with a cantilever with spring constant of 2.8 N/m. The cell body

regions that display higher - than - average values of the elastic modulus correlate with regions with high concentration of actin. The location of the regions with high actin concentration does not change significantly during the AFM measurements.
